# Epigenetic Control of Plant Response to Heavy Metals

**DOI:** 10.3390/plants12183195

**Published:** 2023-09-07

**Authors:** Elisa Fasani, Gianluigi Giannelli, Serena Varotto, Giovanna Visioli, Diana Bellin, Antonella Furini, Giovanni DalCorso

**Affiliations:** 1 Department of Biotechnology, University of Verona, 37134 Verona, Italy; elisa.fasani@univr.it (E.F.); diana.bellin@univr.it (D.B.); 2 Department of Chemistry, Life Sciences and Environmental Sustainability, University of Parma, 43124 Parma, Italy; gianluigi.giannelli@unipr.it (G.G.); giovanna.visioli@unipr.it (G.V.); 3 Department of Agronomy, Food, Natural Resources, Animals and Environment, University of Padua, 35020 Legnaro, Italy; serena.varotto@unipd.it

**Keywords:** plant epigenetics, heavy metal tolerance, potentially toxic elements, plant growth promoting bacteria

## Abstract

Plants are sessile organisms that must adapt to environmental conditions, such as soil characteristics, by adjusting their development during their entire life cycle. In case of low-distance seed dispersal, the new generations are challenged with the same abiotic stress encountered by the parents. Epigenetic modification is an effective option that allows plants to face an environmental constraint and to share the same adaptative strategy with their progeny through transgenerational inheritance. This is the topic of the presented review that reports the scientific progress, up to date, gained in unravelling the epigenetic response of plants to soil contamination by heavy metals and metalloids, collectively known as potentially toxic elements. The effect of the microbial community inhabiting the rhizosphere is also considered, as the evidence of a transgenerational transfer of the epigenetic status that contributes to the activation in plants of response mechanisms to soil pollution.

## 1. Introduction

All living organisms, and all the more, plants as sessile organisms, face the need to overcome challenges provided by hostile environments. Responses and adaptation to environmental stresses take advantage of a variety of strategies, from transitory ones, as the regulation of genic expression or epigenetic modifications, to heritable ones as genetic mutations. The presence of potentially toxic elements (PTEs) is an increasingly diffused environmental condition that affects a significant number of habitats. PTEs, formerly indicated with the term heavy metals, are a class of elements that have a negative biological activity on living organisms when exceeding relatively low levels [1]. Although PTEs are natural components of metalliferous rocks and soils, their widening distribution in the environment is mostly due to mining, industrial activities, fertilizers, and land application of treated wastewater and domestic effluents [2,3]. Contamination by PTEs has received attention worldwide as a major environmental concern, particularly on cultivated lands, considering both the impact on crop fitness and productivity and the introduction of heavy metals in the food chain and the consequent damage on human health [3,4]. The bioavailability of these elements in soils is strongly related to the physico-chemical characteristics of the substrate, as well as to the biological components of the rhizosphere and its interaction with soil and plants [5,6]. PTEs are conventionally divided in two groups: (i) essential elements for living organisms, with a pivotal role in growth and development (e.g., Cu, Fe, Ni, Zn), whose deficiency and excess can both lead to physiological stress due to nutritional imbalance and toxicity, and (ii) non-essential elements (e.g., As, Cd, Hg) that do not have a recognized physiological function and are toxic even at low concentrations [7]. 

**Figure 1:**
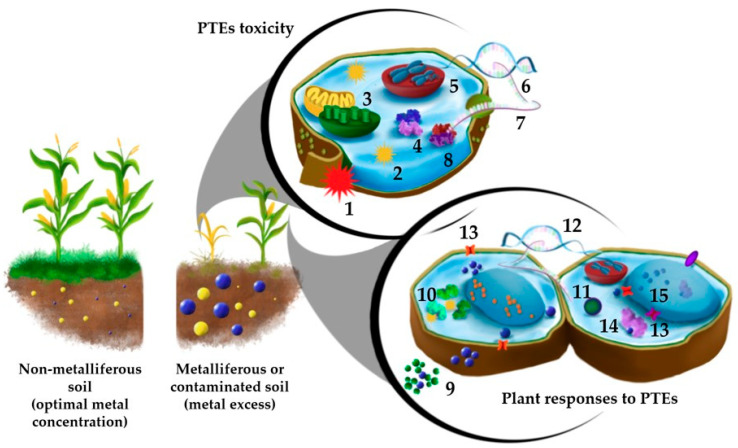
Main mechanisms involved in PTE toxicity and plant responses. Upon metal excess, the cells undergo a variety of injuries that have been highlighted: 1. membrane damage, 2. oxidative burst, with production of ROS, 3. mitochondrial and chloroplast damage, 4. inhibition of enzymes activity, 5. DNA damage, 6. transcription inhibition, 7. protein synthesis inhibition, and 8. protein degradation and denaturation. Plants respond to the excess PTE by activating an array of strategies, both preventing metal entrance into cells (9. metal binding to cell wall and root exudates) and contrasting the metal stress (10. ROS scavenging mechanisms) and decreasing the metal concentration in the cytosol. The latter strategy starts with the sensing and signalling of the metal stress (11. hormone signalling and transduction pathways activation) that eventually promote transcription of stress responsive genes (12), encoding metal ions transporters (13), and proteins involved in PTE detoxification, such as metal chelation (14) and vacuolar sequestration (15).

In biological systems, the excess of essential trace elements or the presence of non-essential metal(loid)s has a consistent impact on cellular, developmental, and physiological processes. When above toxicity levels, PTEs alter the functionality of cell membranes and other cellular components and hamper normal metabolic processes by disturbing protein structure, displacing essential metals in biomolecules or as cofactors, interfering with functional groups, and generating reactive oxygen species (ROS) [8,9,10] (Figure 1). As a result, plants growing in metal-polluted sites may show metabolic disorders and alterations of physiological and biochemical processes, with a consequent reduction in growth and biomass production, as well as abnormal metal accumulation in different tissues and organs [11]. 

However, in naturally metalliferous or historically polluted habitats, plants have evolved strategies to cope with high external PTE concentrations. On the one hand, many species adopt an avoidance mechanism to restrict the uptake of toxic metals and limit their movement in plant tissues. Physical barriers, like thick cuticles and cell walls, selective transport across the plasma membrane, and secretion of root exudates to alter metal availability contribute to this strategy [12,13]. If, on the other hand, PTEs are allowed to cross these barriers, plants respond with tolerance mechanisms, mostly by preventing interactions between metals and vital cellular components and by restoring damages. Among these mechanisms, the production of chelating agents (e.g., organic acids, nicotianamine, and glutathione) and the compartmentalization away from the cytoplasm contribute to detoxify metals [10,14]. Furthermore, the activation of redox detoxification and signalling pathways, including also hormones such as jasmonic acid, ethylene, and salicylic acid, are involved in plant tolerance by coordinating defence responses and mitigating potential cellular damages [10,15,16,17] (Figure 1). 

Plant adaptive processes to different edaphic contexts have led to a wide variety in PTE tolerance and accumulation capacity. Among them, is the unique evolution of the hypertolerance and hyperaccumulation traits found in both angiosperms and pteridophytes [18,19]. Hyperaccumulator plants are a small group of metallophyte or pseudo-metallophyte species able to take up, translocate, and accumulate PTEs in their above-ground organs up to extremely high levels without evident symptoms of toxicity [20,21]. Despite the modest frequency of this phenotype (only ca. 0.2% of all angiosperms, [20]), hyperaccumulator species have allowed the exploration of molecular mechanisms controlling metal tolerance and accumulation. The strategies applied by tolerant species to cope with metal-rich environments mostly rely on the fine tuning of the above-mentioned mechanisms, such as metal transport and chelation or stress response, that are indeed common to all plant species. Although mutations altering protein function or selectivity have been reported, most evidence in this context points to significant changes in the expression of determinant genes, due to combinations of copy number expansion and different transcription regulation [22].

In addition to genetic variations, which were found to be linked with the evolution of tolerance/accumulation traits, all the strategies mentioned above are modulated precisely by epigenetic mechanisms, allowing plants to fit the specific habitats they grow in. Epigenetic modifications, i.e., processes producing changes in DNA activity without altering its nucleotide sequence, include the methylation of cytosine residues within DNA, and the post-translational modification of lysine and arginine residues in histone proteins. These modifications usually affect DNA condensation and accessibility to DNA-binding and regulatory proteins and components of the transcriptional machinery, eventually altering transcription patterns. It is worth noting that epigenetic regulation is a flexible mechanism for plants coping with harsh environments that potentially targets both transient responses and heritable traits [23]. It is easily adaptable in relation to all habitat units, including the interaction with biotic components [24], thus contributing to plant integration in a complex adaptive network. 

The present review focuses on the epigenetic processes in plants, their occurrence during the response to metal stress and the methods available to study them. Space is given particularly to the role of epigenetics in the acquisition of tolerance to PTEs, while also highlighting the effect of the interaction between plants and microorganisms in the rhizosphere of metal-rich soils. 

## 2. Plant Epigenetic Mechanisms: How Do They Work and How Can We Study Them?

Gene transcription and genome stability are controlled by epigenetic mechanisms and chromatin dynamics in the nucleus. 

### 2.1. Histone Modifications

In Eukaryotes, the basic unit of chromatin is the nucleosome. About 146 DNA base pairs are wrapped around each nucleosome core particle that is made up of a histone protein octamer. Four canonical histones, namely, histone H2A, H2B, H3, and H4, are present twice in each nucleosome. Histones are highly conserved globular proteins possessing N-terminal tails which protrude from the surface of the nucleosome octamer and can be covalently modified by methylation, acetylation, phosphorylation, and many other modifications [25]. Eukaryotic genomes also encode for non-allelic histone variants: while canonical histones are usually deposited into nucleosomes during the S-phase of the cell cycle, histone variants can also be integrated during the entire cell cycle [26]. Histone variants can affect nucleosome stability and organization, thus acting on chromatin accessibility and transcriptional outputs. There are many enzymes that add (called writers), bind (called readers), or remove (erasers) histone modifications and that together establish chromatin states, which characterize different genomic regions [27]. In plant chromatin, mono-, di-, and trimethylation of histone H3 at lysine in position 9 (H3K9) and 27 (H3K27) are associated with gene silencing and constitutive or facultative heterochromatin. On the other hand, H3K4 and H3K36 methylation have been shown to either promote or repress transcription, depending on the number of methyl groups that are added [28]. Enrichment in histone acetylation is usually associated with active chromatin in transcribed regions, while histone deacetylation characterizes silenced gene sequences and compacted chromatin [29]. Additionally, chromatin can be remodelled by ATP-dependent chromatin-remodelling complexes (CRCs), which influence histone–DNA interactions, providing accessibility to transcriptional regulators [30]. 

### 2.2. DNA Residue Modifications

In the plant cell, DNA can also be directly modified. In particular, methylation at the 5′ position of cytosine can occur in specific sequence contexts (CG, CHG, or CHH, where H can be an A, T, or C). DNA methylation is considered an epigenetic mark because patterns of symmetrical (CG) DNA methylation are faithfully inherited through DNA replication. Maintenance of plant DNA methylation in CG is catalysed by METHYLTRANSFERASE 1 (MET1), which is recruited by VARIANT IN METHYLATION (VIM) proteins to hemimethylated strands following DNA replication and methylates the unmodified cytosine in the newly synthetized daughter strands. Two DNA methyltransferases, CHROMOMETHYLASE 2 and 3 (CMT2 and CMT3), are responsible for the maintenance of CHG methylation in *A. thaliana*. In plants, CHG methylation and H3 histone modification lysine dimethylation (H3K9me2) reinforce each other through a regulatory feedback loop. Indeed, CMT2 and CMT3 chromo domains bind to H3K9me2-containing nucleosomes in order to target DNA methylation [31]. 

CHH asymmetric methylation is maintained by DOMAINS REARRANGED METHYLTRANSFERASE 2 (DRM2) or CMT2, depending on the genomic region. Through the RNA-dependent DNA-methylation pathway (RdDM), a small RNA (sRNA)-mediated pathway, DRM2 maintains CHH methylation at specific target regions, such as transposons and repetitive elements, both in euchromatin and heterochromatin. By contrast, CMT2 catalyses CHH methylation at histone H1-containing heterochromatin, where RdDM is inhibited. Methylation by CMT2 is impaired by mutations in DECREASED DNA METHYLATION 1 (DDM1), which is a chromatin-remodelling protein that is also required for maintaining DNA methylation in symmetric cytosine sequence contexts [32]. In plant genomes, DNA methylation on repeat sequences and transposons is essential for suppressing transcription and is typical of heterochromatic domains. Consequently, mutations that abolish or alter most DNA methylation lead to transposon activation and are lethal in the majority of plant species [33]. 

Besides the above-described methylation processes, DNA methylation landscapes are dynamically regulated at the genomic level also by demethylation processes. Plants possess a mechanism for active DNA demethylation through DNA glycosylases. These enzymes excise 5-meC and activate its replacement with unmodified C through a base-excision-repair mechanism, which involves numerous proteins and regulatory factors. Active DNA demethylation counteracts excessive methylation in different genomic regions, such as repetitive sequences and transposable elements, avoiding methylation spreading in euchromatin. Plant 5-meC DNA glycosylases are involved in many developmental processes and responses to a variety of biotic and abiotic environmental stimuli [31]. 

### 2.3. Non-Coding RNAs 

Although non-coding RNAs, including microRNAs (miRNA), small interfering RNAs (siRNA), and large non-coding RNAs (lncRNA), are not directly considered as epigenetic modifications, increasing evidence indicates that they are involved at different levels in regulating the epigenetic landscape. In particular, siRNAs are key players in de novo DNA methylation through the RNA-directed DNA-methylation (RdDM) process by directing the methylation machinery to target sequences [31]. Also, lncRNAs have a role in epigenetic regulation: for example, in recruiting enzymes and complexes for histone modification and chromatin remodelling [34]. Finally, although miRNAs regulate gene expression mainly by post-transcriptional processes, some miRNAs were found either to directly drive DNA methylation or to trigger secondary siRNA production by cleavage of target transcripts, thus participating in the RdDM process [35].

### 2.4. Experimental Approaches to Study Epigenetic Regulation

Increasing evidence has indicated that epigenetic modifications are associated with changes in gene expression in response to a variety of stresses, including PTEs [36,37]. Different approaches can be used to investigate how epigenetic marks and/or the epigenome mediate transcriptional regulation in response to heavy metals. Such strategies have benefitted from the advances in next-generation sequencing (NGS) technologies of the last decade. Thanks to these, it has become possible to analyse genome-wide chromatin and DNA methylation states in plant tissues, in plant populations, and across different plant species. NGS technologies have allowed us to determine the epigenomic landscapes and their variations in the model species *Arabidopsis thaliana*, as well as other plants such as rice and tomato, for which reference genomes have been made available. Additionally, the determination of chromatin states provides insight into both the epigenomic features of cis-regulatory elements and genetic variants which affect gene expression and phenotypic traits. Through the integration of chromatin accessibility, DNA methylation, and transcriptome datasets, it is possible to construct comprehensive epigenome landscapes for the annotation of functional elements and for a better understanding of transcriptional regulation during plant development and in response to environmental cues. 

The dynamic modifications of chromatin in plant tissues can be studied by using histone-modification-specific antibodies in chromatin immunoprecipitation (ChIP). Once a specific modification of chromatin and/or DNA is isolated, ChIP-seq (NGS) methods can be applied for genome-wide studies [38]. Despite these significant advancements in the studies of plant chromatin states, the construction of comprehensive epigenome maps for different tissues and genotypes is still limited in non-model species due to the low efficiency of ChIP-seq experiments in plants [39]. However, ChIP assay and the deriving data are still the primary method to investigate protein–chromatin interactions, at least in model species, also in reference to stress-related contexts. For example, in a very recent study, publicly available ChIP-seq datasets of Arabidopsis wild-type and mutants defective in key enzymes of histone modification and chromatin remodelling were used to produce a comprehensive list of iron homeostasis genes with differential enrichment of various histone modifications [40]. Furthermore, novel procedures are being developed to overcome some of the limitations, such as the relatively high amount of plant material needed for chromatin extraction and immunoprecipitation [41]. 

As for direct DNA modification, bisulphite treatment is the most used method for identifying 5-methyl cytosine. Sodium bisulphite induces non-methylated cytosine deamination and converts it to uracil; conversely, the methylated cytosine remains intact. Uracil is finally converted to thymine after amplification through the polymerase chain reaction. In principle, the whole genome bisulphite sequencing (WGBS or BS-Seq) covers all the cytosine information once the data are mapped back to the reference genome [42]. In addition to this technique, novel third-generation sequencing technologies, such as PacBio SMRT and Nanopore sequencing can directly detect DNA methylation in plant genomes and, by unambiguous mapping of repetitive and complex regions of the genome, help to overcome the limitations associated with the fragmented information obtained with short-read sequencing [43]. A detailed description of bioinformatic tools and pipelines designed to map and analyse the datasets in order to answer specific biological questions can be found in other papers [44,45,46,47].

## 3. Epigenetic Mechanisms in Plant Interaction with PTEs

### 3.1. PTE-Induced Modulation of Modifying Enzymes

**Figure 2:**
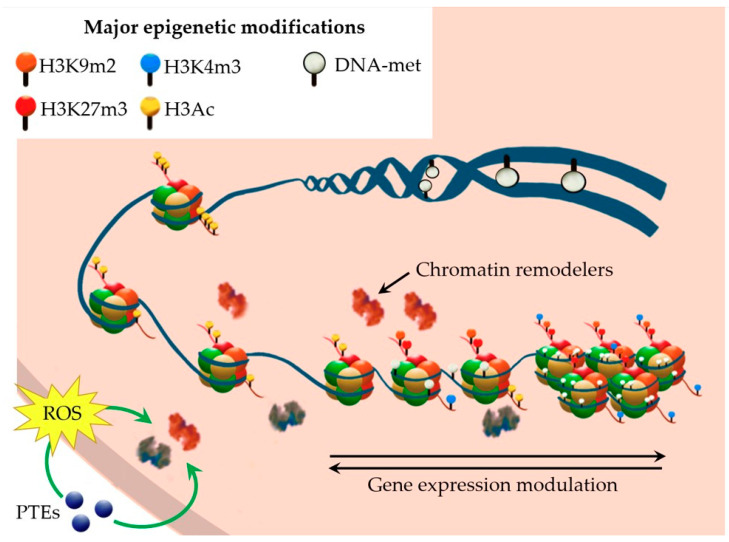
Main epigenetic modifications involved in modulation of gene expression upon PTE stress.

Epigenetic mechanisms play a pivotal role in the response to stresses and the adaptation to adverse environmental conditions. In the last decade, evidence of epigenetic modulation has been accumulating concerning heavy metal stress. Transcriptomic analyses upon metal(loid) treatments have highlighted the selective modulation of genes involved in DNA methylation and demethylation [48,49,50], in histone modification and chromatin remodelling [49,51,52]. Furthermore, the overexpression or mutation of genes involved in epigenetic modifications was proved to alter plant tolerance to PTEs [53,54,55,56], further supporting the involvement of these mechanisms in metal tolerance and accumulation. For example, in *A. thaliana*, the inhibition of DNA demethylases ROS1, DML2, and DML3 determined an increase in DNA methylation upon Cd exposure, which in turn strengthened plant tolerance to Cd stress by improving Fe status in the roots [54]. On the contrary, in rice, an increase in Cd tolerance was promoted by the repression of methylase genes, either by mutation or by the application of the methylation inhibitor 5-azacytidine, and the consequently reduced DNA methylation [49,57]. Epigenetic mechanisms were found to be mediated by ROS homeostasis and signalling in a variety of stresses [58], and especially in response to PTEs [59,60] (Figure 2). How ROS function as signalling molecules is still under debate; it has been proposed that ROS may influence the activity of chromatin-remodeller enzymes by changing their post-translational modifications and, therefore, their activity [58]. 

### 3.2. PTE-Induced Chromatin Modifications

Beyond the specific mechanisms producing the epigenetic modifications, these have been observed to happen at two levels in plants exposed to environmental stresses: on a whole-genome scale or targeting single genes with a significant role in stress response and tolerance. At the global level, epigenetic modifications (in the form, for instance, of massive hypo- or hypermethylation) are a response to genotoxicity and genome instability. The latter two events have been widely established among the toxicity symptoms of PTEs, although they remain only moderately analysed in plants. Several metal(loid)s, such as Cd, Cr, As, and Pb, induce damages on DNA by a variety of mechanisms that are mainly ROS-mediated, with effects going from simple mutations to chromosomal aberrations [61,62,63,64]. 

Epigenetic changes acting on chromatin modelling, DNA methylation in the first place, are likely a strategy to avoid genetic instability ensuing from stress, as reported for other abiotic stresses. However, the phenomenon is complex and strongly depends on the plant genotype, the metal considered, and the experimental conditions; indeed, contrasting profiles have been observed in different species and metals. For example, *Trifolium repens* (metal sensitive) and *Cannabis sativa* (metal tolerant) both showed changes in DNA methylation tending toward hypomethylation when grown in soils artificially polluted with Cd, Cr, and Ni [65]; similarly, Cd produced demethylation in *Brassica napus* [66] and *A. thaliana* [67]. On the contrary, Cd treatment resulted in increased methylation in *Posidonia oceanica* [68], *Datura stramonium* [69], *Noccaea caerulescens* [67], and *Hibiscus cannabinus* [70]; hypermethylation was also produced by Cr in *B. napus* [71] and *Zea mays* [72], by Pb in *Raphanus sativus* [73], and by Cu in *Hydrilla verticillata*, the latter in a ROS-dependent manner [74]. 

The protective role of methylation against genotoxicity and genome instability has been proposed to act by reconfiguring chromatin and preventing mobilization of transposable elements upon stress [49,73,75]. On the other hand, this hypothesis fails to explain the variability in behaviour observed between different species also in response to the same genotoxic metal (e.g., Cd producing either hyper- or hypomethylation in different plant genotypes). However, it should be remembered that epigenetic modifications condition transcription and are, therefore, involved in regulating gene expression, a phenomenon that is strongly influenced by the genetic background. 

Indeed, gene-specific changes in DNA methylation or histone modifications have been observed to occur alongside genome-wide ones [49,65,73,76,77]. A variety of targets have been identified for these changes, which are associated with the modulation of gene expression: interestingly, these include genes involved in cell cycle [78], xenobiotic and/or metal transport, stress response [49,70,73,77,79], and small RNA-mediated gene regulation [49]. Deeper investigation has been performed on some genes specifically involved in plant–metal interactions, highlighting how epigenetic mechanisms play a fundamental role in the response to PTEs. For example, rice Mn transporter *OsMTP11* was upregulated by Mn and, to a lesser extent, by Cd, Zn, and Ni; consistently, these treatments induced demethylation at the CG, CHG, and CHH sites of its promoter, as detected by bisulphite DNA sequencing, suggesting a direct involvement of epigenetic modification in the induction of *OsMTP11* upon metal stress. Interestingly, such demethylation is the prerogative of the metal stress since the treatment with NaCl did not drive either DNA demethylation or *OsMTP11* overexpression [80].

Similarly, the plasma membrane transporter *OsZIP1* and the metallochaperone *OsHMP* were both upregulated in rice in response to Cd and, to a lower extent, other metals. Indeed, Cd induced demethylation of both DNA and histones in the coding sequence of *OsZIP1* and in the promoter of *OsHMP*; the two events were found to correlate with the increased gene expression upon Cd stress [81,82]. In wheat, linked hypomethylation of the promoter regions and increased expression levels were detected in response to Cd, Pb, and Zn for genes encoding the transporters *TaABCC*s and *TaHMA2*, deputed to metal vacuolar sequestration and export to the apoplast, respectively. Interestingly, this was specifically observed in a metal-tolerant genotype, compared with a sensitive variety [83]. 

### 3.3. Transgenerational Memory of the PTE Stress Response

The major advantage of using epigenetic modification to modulate plant response to heavy metals or, more in general, stress is that of the extreme flexibility of the mechanisms that do not require variation in the DNA sequence [84,85]. On the one hand, transient chromatin modifications allow for reversion upon the end of the stress, providing fast adaptation and response to changing environments. Although, in the case of PTE excess, this situation is unrealistic in nature, transience in epigenetic markers was demonstrated for phosphate starvation and subsequent resupply in Arabidopsis [86]. In laboratory conditions, some experiments indicate that reversion to the original epigenetic status happens, at least partially, upon recovery from metal stress [59,87] or in the following untreated generations [48,88]. On the other hand, the same experiments on multiple generations demonstrated transgenerational memory of epigenetic mechanisms in PTE stress. Indeed, epigenetic mechanisms were proposed as underlying transgenerational memory of metal tolerance in Arabidopsis [89]. Interestingly, such transgenerational memory of the stress information is transferred to the immediate progeny, but this transmission ends when the following generations are grown in the lack of the metal stress [89]. Conversely, in rice, changes in DNA methylation induced by Hg were reverted only for less than 50% in the first following generation, the remaining being composed by either conserved patterns or further new changes. The second generation also showed extremely high levels of epigenetic conservation [48], together with conserved changes in gene expression of determinant genes [88]. Similarly, Cu-induced epigenetic changes in *Trifolium repens* were conserved both after transfer of the mother plant to non-polluted soil and, mostly, in the clonal progeny derived [90]. 

### 3.4. Epigenetic Modifications in the Evolution of PTE-Tolerant Species

The transgenerational memory of epigenetic changes is, most likely, a driving force behind plant adaptation to adverse environmental conditions, together with actual changes in DNA sequence.

As previously described, PTE effects on global chromatin organization and DNA epigenetic modifications are extremely variable, depending on both the chemical element considered (including its biological role or lack thereof) and the genotype of the plant considered. In fact, differences are very evident when directly comparing non-tolerant vs. tolerant species. For example, non-tolerant *A. thaliana* had a higher basal methylation level than hyperaccumulator *N. caerulescens* and reacted to Cd treatment with DNA demethylation, whereas *N. caerulescens* showed an increased global methylation level [67]. Even at the level of population or cultivar, epigenetic-mediated adaption to metal-polluted soils was demonstrated. The comparison between two Cd-tolerant populations of *Arabidopsis halleri* with different edaphic strategies, one a Cd excluder (I16) and the other a hyperaccumulator (PL22), evidenced contrasting behaviour in Cd-induced DNA methylation, interpreted as a potential defence mechanism to regulate chromatin condensation and prevent nuclear damages [91]. Interestingly, *Armeria maritima* populations from polluted and unpolluted soils, genetically equidistant, showed clustering of metallicolous populations when DNA methylation was considered: even though the metal-tolerant populations are geographically distant and the result of independent adaptive events, epigenetic changes can be ascribed for a significant percentage to their edaphic character [92]. A role of epigenetic mechanisms in allowing adaptation to PTE-polluted soils was found also in *Chenopodium ambrosioides* [93]; in the same species, significant differences in epigenetic mechanisms were found when comparing Mn-sensitive and Mn-tolerant populations [94]. Different methylation profiles also characterized non-tolerant and Cr-tolerant strains of the green alga *Scenedesmus acutus*; in the specific, when comparing genes involved in the sulphate pathway, putatively involved in Cr tolerance, the methylation differences were consistent with differential gene expression [77]. 

Apart from natural adaptive processes, epigenetic variation and its correlation with PTE tolerance has raised increasing interest in agricultural crops. Cereals in particular have been research targets in this field. In rice, metal-tolerant hybrids showed differential methylation patterns in metal-associated genes both upon Cd, Cr, or Cu treatments and in comparison to sensitive genotypes [95]. A wheat cultivar characterized by metal tolerance showed a distinct hypomethylation in the promoters of target transporter genes that was absent in the non-tolerant variety [83]. Different changes in DNA methylation were also detected in Al-tolerant and Al-sensitive triticale genotypes upon Al treatment [96]. Finally, three Al-tolerant barley cultivars were characterized by the insertion of an LTR retrotransposon in the promoter of *HvAACT1*, encoding a plasma membrane citrate transporter involved in citrate secretion for in-soil Al detoxification. This retrotransposon induces higher expression of *HvAACT1* and was identified in a wide number of barley accessions, in which Al tolerance was directly associated with the degree of LTR methylation and consequent regulation of *HvAACT1* expression levels [97]. This evidence has suggested the possibility to act on the epigenome to modulate crop tolerance to PTEs, although research in this field has as yet concentrated on cultivar characterization, rather than actual strategies for epigenetic control. 

## 4. Novel Trends in the Epigenetic Control of Plant Response to PTEs: The Role of Biotic Components of the Rhizosphere

Plant-growth-promoting bacteria (PGPB) are plant-associated, free-living soil-borne bacteria which can enhance plant growth through various mechanisms. PGPB stimulate plant growth through the production of phytohormones, the increase of soil nutrients bioavailability, and controlling plant pathogens and diseases by the production of antimicrobial molecules [98]. Arbuscular Mycorrhizal Fungi (AMF) are soil-borne fungi that can significantly improve plant nutrient uptake and resistance to several abiotic stress factors. The fungal mycelium colonizes the roots of many plants even belonging to different species, resulting in a common mycorrhizal network which increases resistance to invasive plants, tolerance to abiotic stress, and fungal-mediated transport of phosphorus (P) and nitrogen (N) to plants [99]. 

### 4.1. PTE Tolerance in Plants Interacting with PGPB and AMF

PGPB and AMF are known to exert beneficial effects on plants exposed to PTEs. Considering this point of view, rhizosphere microorganisms can contribute either to enhance metal bioavailability, increasing plant metal accumulation, or, on the contrary, to stabilize PTE ions in the soil matrix, preventing or reducing root absorption and root-to-shoot translocation of excess PTEs. These abilities can be attributed to the microbial metabolism and processes such as precipitation, biotransformation (e.g., methylation, volatilization, oxidation or reduction, formation of complexes), and adsorption of ions onto the microbial structures. Although there is wide evidence of the positive role of PGPB and AMF in increasing PTE tolerance and accumulation in plants (especially in hyperaccumulator species), to date, little is known regarding the effect of soil microorganisms in modulating plant gene expression or epigenomes in response to PTEs. In some works, expression modulation mediated by PGPB has been observed in association with greater plant tolerance to metals in contaminated sites. Genes that are modulated by PGPB association encode metal transporters or participate in the oxidative stress response, contributing to plant tolerance towards PTEs (see Table 1 for an overview). 

For instance, *Pseudomonas aeruginosa* and *Burkholderia gladioli* were able to downregulate metal transporters in *Lycopersicon esculentum* seedlings grown upon Cd stress, reducing metal uptake and bioavailability and, therefore, promoting Cd tolerance and growth [100]. Similarly, *Pseudomonas fluorescens* upregulated *AtPCR2*, which encodes a membrane protein involved in metal transport and detoxification, enhancing Cd resistance in inoculated plants [101]. Once inoculated in Arabidopsis, *Bacillus amyloliquefaciens* (strain SAY09) promoted the expression of genes involved in sensing Fe status and in Fe transport, e.g., *FIT1*, *IRT1*, and *FRO2*, under excess Cd, enhancing plant tolerance by limiting Cd-induced Fe deficiency [102]. As for genes related to the PTE-induced oxidative stress, inoculation with strains of *Bacillus pumilus* and *Bacillus firmus* improved the tolerance of *Solanum tuberosum* to Zn by enhancing the expression of a variety of ROS-scavenging enzymes (e.g., *APX*, *SOD*, *CAT*, *DHAR*, and *GR*) [103]. Similar results were reported when inoculating *Lathyrus sativus* with a PGPB consortium prior to the application of excess Pb [104]. PGPBs can also increase the phytoextraction capacity by modulating the expression of specific genes in hyperaccumulator species. For instance, endophytic strains of *Pseudomonas fluorescens* enhanced the accumulation of Cd and micronutrients, such as Fe, Zn, and Cu, in the Zn/Cd hyperaccumulator *Sedum alfredii*, by regulating the expression of a variety of genes involved in Cd uptake, translocation to the shoot, and allocation into vacuoles, (e.g., *ZIP transporters*, *IRT1*, *HMA2*, and *HMA3*) [105,106]. 

### 4.2. Plant Epigenetic Regulation and Rhizosphere Microorganisms

Although evidence is still limited, the interaction between plants and the associated beneficial microorganisms seems to also influence the epigenetics of plant response to PTEs. Data obtained by epigenomics analysis are timidly emerging, shedding light on the dynamic of DNA methylation, histone modification, and small non-coding RNAs’ modulation induced by PGPB or AMF on PTE-treated plants. For instance, the patterns and temporal changes of DNA methylation were analysed in leaves of *Populus alba* plants to determine the combined effect of inoculation with the AMF *Funneliformis mosseae* and *Rhizophagus intraradices* and stress due to excess Cu and Zn. Interestingly, extensive DNA hypomethylation occurred only in mycorrhized plants after 6 months of Cu and Zn excess, pointing to an important role of epigenetic mechanisms in the long-term adaptation. Furthermore, the response induced by PTEs and AMF was found to spread systemically from roots to shoots [107]. Moreover, differences in methylation were detected in genes involved in ROS scavenging, RNA processing, cell wall constitution, and amino acids metabolism, all mechanisms that may confer improved stress tolerance to the colonized plants [107]. A similar effect on methylation was detected in *Arundo donax* inoculated with the PGPBs *Stenotrophomonas maltophilia* and *Agrobacterium* and upon Se stress [108]. 

Recently, small RNAs have been shown to play important roles in cross-kingdom communication, notably in plant–pathogen relationships, and suppression of plant miRNAs by PGPB results in enhanced plant defence against pathogens [109]. In the rhizosphere environment, the interaction between plants and microorganisms is shaped by a variety of exchanged signals, likely including sRNAs [110]. Although evidence has been found on sRNA inter-kingdom exchanges by extracellular vesicles released in the medium, the focus has been mostly on plant–pathogen interactions [111]. On the other hand, no direct evidence has been reported so far regarding the role of sRNAs in modulating the plant–rhizobiome interaction and the epigenetic response of both in the presence of excess PTEs. 

Transgenerational epigenetic inheritance due to modifications induced by the rhizosphere microorganisms is another unexplored topic. Interestingly, the ability of AMF and PGPB to induce epigenetic changes transmitted to the progeny has been reported. For example, in *Geranium sylvaticum*, the AMF status of father plants can affect DNA methylation in seeds, and these changes in DNA methylation are further dependent on the gender (i.e., female or hermaphrodite) of the genotype adopted as female counterpart [112]. As for PTE–plant–microorganism interaction, research is still in its early stages. In the view of increasing phytoremediation potential, it was demonstrated that inoculation with *Arthrobacter* (strain PGP41) enhanced plant fitness upon Cd stress in the North American Mn/Cd hyperaccumulator *Phytolacca americana*, thanks to a variety of growth-promoting features as well as the ability to secrete organic acids in a Cd-dependent manner [113]. Recently, this PGPB, as well as a newly identified *Bacillus* strain, was found to induce long-term changes in root gene expression and epigenome when inoculated in *P. americana* plants grown in unpolluted soil. Interestingly, the observed variations were also conserved after the elimination of the bacteria; differential methylation correlated with the modulation of gene expression and was necessary for the growth-promoting activity of the inocula [114]. These preliminary results offer new strategies for microbiome manipulation to promote plant growth in PTE-contaminated soils through the application of PGPB or AMF.

## 5. Conclusions

Epigenetic modifications play a pivotal role in the control of PTE stress in plants. PGPB and AMF inhabiting the rhizosphere also contribute to modulate the epigenetic status of the plant they interact with, and probably are likewise involved in inheritance of the epigenetic modifications. The increasing understanding of these processes opens novel perspectives that may help to improve plant adaption and fitness when growing in metal-rich soils. However, more efforts should be taken to identify processes allowing for controlled epigenetic changes that can be applied to crops. Among them, microorganism-induced modifications should be further investigated, for example, by uncovering whether specialized molecules, metabolites, and other signalling molecules, such as nucleic acids fragments (miRNA) are produced by PGPB/AMF or by plants.

## Figures and Tables

**Table 1:** Summary of studies showing the effect of PGPB on the expression of plant genes involved in PTEs response. ↑ = increase in gene expression; ↓ = decrease in gene expression.

PGPB	Plant Species	PTE	Modulated Genes	Reference
*Pseudomonas aeruginosa* *Burkholderia gladioli*	*Lycopersicon esculentum*	Cd	↓ members of the P-type ATPase metal transporter family	[100]
*Pseudomonas fluorescens*	*Arabidopsis thaliana*	Cd	↑ *AtPCR2*	[101]
*Bacillus amyloliquefaciens* SAY09	*Arabidopsis thaliana*	Cd	↑ *FIT1*, *IRT1*, *FRO2*	[102]
*Bacillus pumilus* *Bacillus firmus*	*Solanum tuberosum*	Zn	↑ *APX*, *SOD*, *CAT*, *DHAR*, *GR*	[103]
*R. leguminosarum* M5 + *P. fluorescens* K23 + *Luteibacter* sp. + *Variovorax* sp.	*Lathyrus sativus*	Pb	↑ *GR*, *GST*	[104]
*Pseudomonas fluorenscens* Sasm05	*Sedum alfredii*	Cd	↑ *SaHMA2*, *SaHMA*, *ZIP2*, *ZIP3*, *ZIP4*, *ZIP11*, and *IRT1*	[105]
*Pseudomonas fluorenscens* SaMR12	*Sedum alfredii*	Cd	↑ *SaIRT1*, *SaZIP1*, *SaZIP3*, *SaHMA2*, *SaHMA3*, *SaNramp1*, *SaNramp3*, *SaNramp6*	[106]

## Data Availability

No new data were created or analysed in this study. Data sharing is not applicable to this article.
